# Genomic variation in *Plasmodium vivax* malaria reveals regions under selective pressure

**DOI:** 10.1371/journal.pone.0177134

**Published:** 2017-05-11

**Authors:** Ernest Diez Benavente, Zoe Ward, Wilson Chan, Fady R. Mohareb, Colin J. Sutherland, Cally Roper, Susana Campino, Taane G. Clark

**Affiliations:** 1 London School of Hygiene and Tropical Medicine, Keppel Street, London, United Kingdom; 2 The Bioinformatics Group, School of Water Energy and Environment, Cranfield University, Cranfield, Bedfordshire, United Kingdom; 3 Department of Pathology & Laboratory Medicine, Diagnostic & Scientific Centre, Faculty of Medicine, University of Calgary, Calgary, Alberta, Canada; Université Pierre et Marie Curie, FRANCE

## Abstract

**Background:**

Although *Plasmodium vivax* contributes to almost half of all malaria cases outside Africa, it has been relatively neglected compared to the more deadly *P*. *falciparum*. It is known that *P*. *vivax* populations possess high genetic diversity, differing geographically potentially due to different vector species, host genetics and environmental factors.

**Results:**

We analysed the high-quality genomic data for 46 *P*. *vivax* isolates spanning 10 countries across 4 continents. Using population genetic methods we identified hotspots of selection pressure, including the previously reported *MRP1* and *DHPS* genes, both putative drug resistance loci. Extra copies and deletions in the promoter region of another drug resistance candidate, *MDR1* gene, and duplications in the Duffy binding protein gene (*PvDBP*) potentially involved in erythrocyte invasion, were also identified. For surveillance applications, continental-informative markers were found in putative drug resistance loci, and we show that organellar polymorphisms could classify *P*. *vivax* populations across continents and differentiate between *Plasmodia spp*.

**Conclusions:**

This study has shown that genomic diversity that lies within and between *P*. *vivax* populations can be used to elucidate potential drug resistance and invasion mechanisms, as well as facilitate the molecular barcoding of the parasite for surveillance applications.

## Background

The *Plasmodium vivax* malaria parasite is the second most virulent malaria species after *P*. *falciparum*. Geographically, it is found throughout Asia, South and Central America, Oceania, Middle East and some parts of Africa, with nearly 2.85 billion people at risk of infection **[1].** Although *P*. *vivax* contributes to almost half of all malaria cases outside Africa, as it kills infrequently and is not amenable to continuous *in vitro* culture, it has been relatively neglected compared to the more deadly *P*. *falciparum* **[2].** However, as *P*. *vivax* drug-resistant strains emerge and spread and fatality rates increase, the need to implement better control and elimination strategies is becoming urgent. Many of the interventions used for controlling *P*. *falciparum* malaria are not as effective against *P*. *vivax*. Consequently, *P*. *vivax* has become the dominant malaria parasite in several countries where *P*. *falciparum* transmission has been successfully reduced. Hence, control and elimination of *P*. *vivax* malaria calls for additional interventions, notably against the dormant liver stage of the parasite. However, gaps in our knowledge of *P*. *vivax* epidemiology and biology may compromise its control. Genomic research can contribute greatly to enhancing our understanding of *P*. *vivax* basic biology and evolutionary history, supporting the development and surveillance of new interventions.

Since the first characterisation of the *P*. *vivax* genome sequence (Sal-1, **[3]**), several population genetic studies, based on microsatellite data and more recently using whole genomes, have shown that this parasite is more polymorphic than *P*. *falciparum* **[4,5,6,7]**. *P*. *vivax* populations harbour high genetic diversity, even on small spatial scales, and can differ extensively between locations due to vector species, host genetics and environmental factors **[4,5,6]**. Genetic variation enables the parasite to overcome host immune responses and antimalarial drugs to establish persistent infections and increase transmission. Genomic studies in natural populations of *P*. *vivax* can pinpoint genetic regions that are under selective pressure, including those associated with resistance to antimalarial drugs. Such studies can also contribute to the identification of vaccine targets. Moreover, global genomic studies can assist with identifying sets of polymorphism private to populations, allowing the monitoring of gene flow over space and time, and the tracking of imported infections. By developing a molecular barcode of individual parasites it will also be possible to distinguish recrudescent from re-infections.

Highly polymorphic microsatellites have been the preferred method of genetic analysis, revealing high levels of diversity and highlighting interesting genotypic patterns and geographical clustering across global populations **[8, 9, 10, 11, 12].** The advancement of whole genome sequencing technologies has opened up opportunities to obtain a comprehensive picture of the epidemiology and structural variation of *P*. *vivax*. There is now the ability to perform genome-wide analysis of the various populations without the need for *in vitro* culture and overcoming difficulties with low parasitaemias and high human DNA contamination **[13].** Recent studies using genome-wide SNPs highlighted that signals of natural selection suggest that *P*. *vivax* is evolving in response to antimalarial drugs and is adapting to regional differences in the human host and the mosquito vector **[6,7].** Several other whole genome sequence studies have been published **[13, 14, 15, 16]**, covering 10 countries. Using these and other data, we explore the genetic diversity within and between continents, identify signatures of drug pressure and molecular barcodes that could be useful for determining the source of infections and monitoring parasite populations.

## Methods

### Samples and sequence data

Publicly available whole genome sequence data for 74 *P*. *vivax* samples were gathered from multiple sources, and included reference strains (India VII, Mauritania X, North Korea II, Brazil I, Sal-1 from El Salvador (see [**2**])), field and clinical isolates (Cambodia (n = 3) [**13**], Thailand (n = 39) [**13**], Madagascar (n = 3) **[2,17]**, Colombia (n = 8) [**14**] and Peru (n = 11) **[7,15]**) and clinical samples from travellers (to Papua Indonesia (n = 2) [**13**], India (n = 2) [**13**], and Papua New Guinea (PNG, n = 6) [**13**]). All sequencing data for non-reference strains were generated using Illumina paired end technologies (read lengths ≥75bp). The raw sequence data were mapped to the Sal-1 reference genome (version 10.0) using *bwa-mem* with default parameters. SNPs (n = 447,232) were identified using the *samtools* software suite (samtools.sourceforge.net) with high quality scores (*phred* score >30, 1 error per 1 kbp). Genotypes were called using ratios of coverage, where the minimal heterozygous calls still present after filtering were converted to the majority genotype if the coverage ratio was 80:20 or greater **[18,19]**. SNPs were retained if they were biallelic, had low genotype missingness (<10%) and heterozygous (<0.4%) calls. SNPs in regions of extreme coverage and very low coverage were excluded, as well as in non-unique regions (using a k-mer approach with length of 54 bp) and highly polymorphic VIR genes. Two samples were found to have *P*. *vivax* and *P*. *falciparum* co-infections (ERR020124 and SRR828528), and were excluded from population genetic analysis. Isolates were retained if they had at least average 10-fold genome-wide coverage, and at most 10% missing genotype calls. The final high quality dataset consisted of 46 (62.2%) isolates (Thailand 22, Southeast Asia 24, South America 11; other 11; **S1 Table**) and 219,288 SNPs, and used as the basis of population genetic analyses. *FreeC* software (http://bioinfo-out.curie.fr/projects/freec/tutorial.html) was used to identify regions of the genome with a significant increase or decrease in read coverage identifying potential copy number variants (CNVs) after accounting for GC bias through coverage normalization. Regions identified as CNVs were inspected visually and assessed using *de novo* assembly methods **[20]**.

### Population genetics

Genetic diversity was estimated using the average pairwise nucleotide diversity (*π*) with the R package *"pegas"*. An in-house R script was used to compute the allele frequency-based Tajima's *D* test **[21]** to identify genes under balancing selection in the individual populations (values > 2.5; **[18]**), this method was chosen over the dN/dS approach given the latter being not fit for analysis on individual populations **[22]**. To detect signals of directional selection, the integrated Haplotype Score (*iHS*) approach **[23]** was applied to individual populations supported by a principal component analysis (PCA). This approach used the most frequent allele where mixed calls where found so the haplotype analysis will be based on the most abundant strain in each sample **[7].** *P*-values for *iHS* were computed from standardised values based on a 2-tailed conversion from a Gaussian distribution **[19]**. The Salvador-I being the reference and oldest sample was used as ancestral haplotype. Multiplicity of infection was estimated using a novel method of counting the unique haplotypes formed by polymorphism on paired sequencing reads (*estMOI*, **[24]**). For comparisons between populations, we first applied PCA and neighbourhood joining tree clustering based on a matrix of pairwise identity by state values. These analyses were followed by applying the cross population long-range haplotype method (*XP-EHH* **[25],** *Rsb* implementation **[19]**) and the population differentiation metric *F*_*ST*_ **[26]**. *P*-values for the *Rsb* estimates were calculated using a Gaussian approximation **[19]**. A significance threshold of *P* < 0.001 was established for both *iHS* and *Rsb* using bootstrap- and permutation-based simulation approaches **[18,19].** We used the ranked *F*_*ST*_ statistics to identify the informative polymorphism for the barcoding of populations and driving the clustering observed in the PCA. Linkage disequilibrium (LD) was assessed in the two populations with the largest sample sizes (Thailand and South America) using the *r*^*2*^ and *D’* metrics **[27]**, calculated for pairs of SNPs with different physical separation up to 2 kbp using a sliding window approach. The SNPs were annotated and effects of variants on genes (such as amino acid changes) were predicted using s*npEFF* software **[28].** The R statistical package was used to analyse SNP data, including implementation of selection analyses using the “rehh” library.

## Results

### Genetic polymorphisms

**Fig 1:**
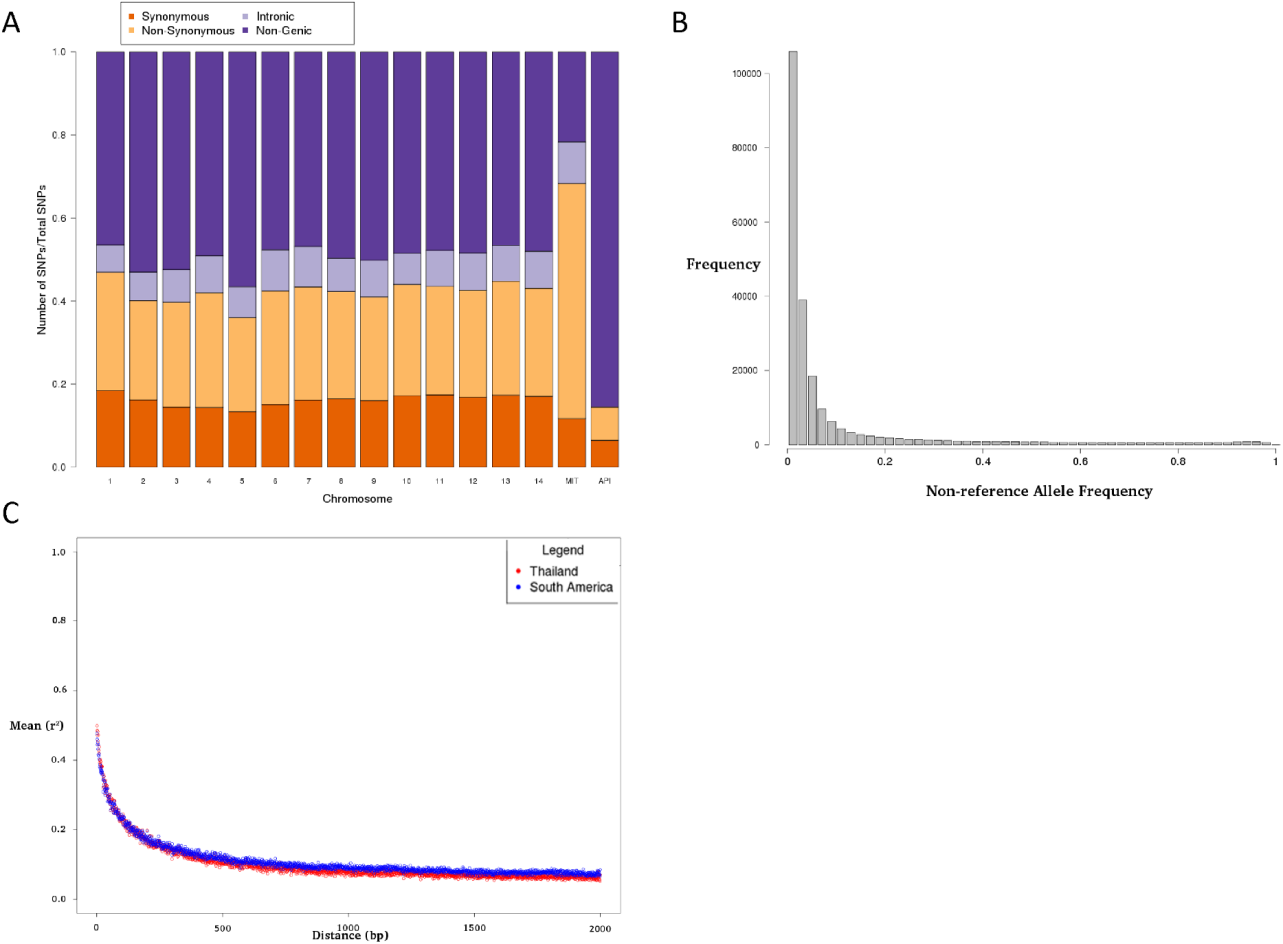
(a) SNP locations by annotation*. (b) Minor allele frequency spectrum indicates a predominance of rare alleles. (c) Linkage disequilibrium (r2) decays rapidly with physical genetic distance. * established using *snpEFF* software.

The genomic coverage in the nuclear genome was high (median 103-fold, range (30-5973-fold), and in keeping with multiple organellar copies, the mitochondria and apicoplast coverage was 30-fold and 1.8 fold greater than the nuclear coverage. The density of SNPs in the nuclear genome (219,288 SNPs, 1 every 99 bp) was greater than in the mitochondrial (23 SNPs, 1 every 165 bp) and apicoplast genomes (176 SNPs, 1 every 165 bp) (**S2 Table**). Although 60% of the annotated reference genome is coding (chromosomal range: 54%-64%), approximately half the SNPs in the isolates were found in genic regions (mean 48% per chromosome, range 43% to 52%) (**Fig 1A**). The proportion of non-synonymous sites is consistent with those found in other *Plasmodium* species, with 52% of coding SNP sites being non-synonymous in the nuclear genome, 36% in the mitochondrion and 56% in the apicoplast. The differences in these genomes suggest they may be subject to differential selective pressure **[29]**. The majority of SNPs are rare, with nearly half of the mutations (45%) being observed in single samples (**Fig 1B**) as seen in other *Plasmodium* populations **[18].** There was some evidence of polyclonality in 22 samples (Cambodia 1/2, Colombia 5/5, Madagascar 2/2, PNG 2/5, Thailand 11/22).

Analysis of structural variants and copy number variants was limited to Thai, Cambodian and Madagascan isolates, which had high and uniform genomic coverage. CNVs were located less than 1 kbp distance from the *MDR1* gene (chromosome 10, *PVX_080100*) in Cambodian and Thai isolates **(S1 Fig**). Several *MDR1* variants have previously been reported, some considered putative chloroquine- and mefloquine-resistance alleles **[14,30–35].** At the *MDR1* locus, we observed either a duplication of ~35kb (position 351kbp to 389kbp, n = 1, Thailand), a major deletion in the promoter region of the gene (n = 7, Thailand; n = 1 Cambodia), or a combination of both structural variants, including two copies, one with the deletion in the promoter and another copy with a complete promoter (n = 4, Thailand); as confirmed by the increase or decrease in coverage and accumulation of split reads in the regions where a break in the coverage occurs. The known duplication in the Duffy binding protein *PvDBP* (chr. 6: 974,000–982,000, *PVX_110810*) in Malagasy **[36]** was confirmed in one of the two Madagascan isolates (SRR828416). The *PvDBP* gene is potentially involved in erythrocyte invasion, and the duplication was also observed in thirteen Thai isolates. A further duplication was observed in *Pv-fam-e* (a RAD gene, chr. 5: 895,000–900,000, n = 8, Thailand), a gene linked to *P*. *vivax* selectivity for young erythrocytes and/or immune evasion **[36].**

### Assessing genetic diversity, LD and positive directional selection

The average polymorphism (pair-wise mismatches measured by nucleotide diversity *π*) was calculated by gene and chromosome. There was little difference across the chromosomes with mean 11.1x10^-4^ (range 6.0 x 10^−4^ to 19.0x10^-4^), which is consistent with other studies with similar sample size **[14]** as well as larger datasets when restricted to high quality SNPs (1.5x10^-3^) **[6, 7].** LD decays rapidly for non-rare polymorphism (minor allele frequency ≥ 5%) within a few hundred base pairs, and reaches a baseline within 500bp in South American and Thai nuclear genomes (**Fig 1C**). Like *P*. *falciparum*, there is a high correlation between non-rare SNPs (median *D*’ 0.918, range 0.425–1) in the mitochondrial and apicoplast genomes supporting the inference that the organelles are co-inherited and supporting the view that these SNPs have potential utility for barcoding **[29].**

To examine evidence for signatures of positive natural selection we calculated the *iHS* metric in the Thailand and South America populations, informed by the population structure reported in **S3 Fig**. Five contiguous loci of strong positive directional selection were identified, including the *MRP1* gene (*PVX_097025*) and its promoter region in Thailand, and a region surrounding the *MRP2* gene (*PVX_097025*, *P*. *falciparum* homologue associated with primaquine and antifolate drug sensitivity **[14]**). *S*everal surface proteins were identified in both populations, including the *MSP7* and *MSP3*.*1*, which are thought to be under selection pressure due to their role in erythrocyte invasion and strong vaccine candidates and have been identified before by other studies using sanger sequencing **[29] (Table 1**, **Table 2, S2 Fig**). In addition, some helicases showed strong signals of selection (*PVX_088190* and *PVX_111220*) which were also detected in the same study **[36]** reinforcing the method used. Furthermore, we identified in South America a proximal region of selection (chr14: 1,414,164–1,479,586) described elsewhere **[7].**

**Table 1:** Regions under directional selective pressure in Thailand *.

**Chr**	**Position / Range**	**Max *iHS***	**Gene**	**Annotation**
**1**	284000	5.083	*PVX_087910*	E3 ubiquitin-protein ligase, putative
**1**	379430	3.511	.	.
**2**	148413	4.319	.	**Promoter region MRP1**
**2**	158122	3.452	***PVX_097025***	**multidrug resistance-associated protein 1, MRP1**
**4**	576773	4.289	.	.
**4**	629852	3.483	*PVX_003770*	merozoite surface protein 5 (MSP5)
**5**	673939	3.566	*PVX_089575*	trafficking protein particle complex protein, TRAPPC2L
**7**	778719	3.986	.	.
**7**	1397181	5.829	*PVX_086903*	Plasmodium exported protein, unknown function
**8**	766604	3.709	*PVX_095055*	Rh5 interacting protein, putative (RIPR)
**8**	921104	3.451	*PVX_095235*	protein phosphatase inhibitor 2, putative
**8**	927191	3.489	*PVX_095245*	hypothetical protein, conserved
**8**	985454	3.778	*PVX_095305*	hypothetical protein, conserved
**9**	107123	6.186	*PVX_090925*	protein kinase domain containing protein
**9**	311594	3.590	.	.
**9**	526557	4.115	*PVX_091440*	hypothetical protein, conserved
**10**	1222646	5.499	*PVX_097715*	hypothetical protein
**10**	1225529	4.109	.	.
**10**	1261650	9.180	.	Promotor region MSP3.1
**10**	1261852	3.982	*PVX_097670*	merozoite surface protein 3 (MSP3.1)
**11**	926166	4.034	.	.
**12**	732115	3.531	*PVX_082735*	thrombospondin-related anonymous protein (TRAP)
**12**	734223–745860	4.901	*PVX_082730*	hypothetical protein, conserved
**12**	746536	4.319	.	.
**12**	751773	5.473	*PVX_082710*	hypothetical protein
**12**	752332	3.558	.	.
**12**	765929	6.849	.	.
**12**	766784	6.170	*PVX_082675*	merozoite surface protein 7 (MSP7)
**12**	864218	3.561	*PVX_082510*	hypothetical protein
**12**	865780	3.930	*PVX_082505*	CPW-WPC family protein, putative
**12**	1020235	5.042	.	.
**12**	2475528	4.740	.	.
**12**	2540841	3.633	*PVX_118270*	serine/threonine protein kinase, putative
**12**	2622092	3.446	*PVX_118345*	protein transport protein SEC7, (SEC7)
**12**	2638874	4.900	.	.
**12**	2671299	3.501	*PVX_118380*	GTP-binding protein, putative
**12**	2732268	3.486	*PVX_118460*	hypothetical protein, conserved
**14**	3028986	5.168	.	.

* |iHS| > 3.

**Table 2:** Regions under directional selective pressure in South America *.

Chr	Position / Range	Max *iHS*	Gene	Annotation
**1**	490369	3.020	*PVX_088190*	helicase, putative
**1**	662401	3.050	*PVX_093585*	SF-assemblin, putative
**2**	244790–1	5.316	.	Promoter region PVX_081315
**3**	247791	3.813	*PVX_000860*	hypothetical protein, conserved
**3**	372679	4.109	*PVX_000695*	hypothetical protein, conserved
**4**	574831	3.029	*PVX_003830*	serine-repeat antigen 5 (SERA)
**5**	560637	3.649	*PVX_089445*	RAD protein (Pv-fam-e)
**5**	1046482	3.358	*PVX_090020*	hypothetical protein, conserved
**6**	627960–1	3.605	*PVX_111230*	hypothetical protein, conserved
**7**	437942–60	4.773	*PVX_099005*	cysteine repeat modular protein 1, CRMP1
**7**	527651	3.182	*PVX_099125*	pseudouridylate synthase, putative
**7**	1116251–2	5.712	*PVX_099915*	RNA-binding protein, putative
**7**	1214179	6.840	*PVX_087145*	nucleolar protein Nop52, putative
**8**	219359	3.180	*PVX_094405*	hypothetical protein, conserved
**9**	730034	3.611	*PVX_091700*	circumsporozoite-related antigen, EXP1
**9**	751857	3.149	.	Promoter region PVX_091715
**9**	829890	3.161	*PVX_091770*	calcium-dependent protein kinase 7, CDPK7
**9**	1042906–7	5.519	*PVX_092035*	6-phosphofructokinase, putative
**9**	1136873	3.110	*PVX_092160*	hypothetical protein, conserved
**10**	380535	3.108	*PVX_080110*	G10 protein, putative
**10**	1063432	3.592	*PVX_097895*	TBC domain containing protein
**10**	1257251	3.291	.	Promoter region MSP3.2
**10**	1260441–2	5.201	.	1 Kb from MSP3.2
**11**	822715	4.026	*PVX_114575*	transmembrane amino acid transporter protein
**11**	1973708	6.074	.	.
**12**	955488	3.652	*PVX_082400*	myosin C, putative
**12**	1317195	3.435	*PVX_116815*	hypothetical protein, conserved
**13**	215135	3.283	*PVX_084350*	hypothetical protein, conserved
**13**	611668	5.708	*PVX_084755*	hypothetical protein, conserved
**13**	856226–30	4.519	*PVX_085030*	aspartyl protease, putative
**14**	1275835	3.114	*PVX_123250*	aquaporin, putative (AQP2)
**14**	1665191	3.572	*PVX_123685*	histone-lysine N-methyltransferase, SET10
**14**	1875833	4.986	*PVX_123890*	hypothetical protein, conserved

* |iHS| > 3.

### Allele frequency spectrum and balancing selection

The allele frequency spectrum of different classes of nucleotide sites all show an excess of rare alleles, with coding, non-synonymous, synonymous and intergenic sites more skewed than expected under a Wright-Fisher model of constant population size **[18].** This observation could indicate a population expansion in the recent past, where as a population grows in size, the frequency of rare alleles also increases **[18]**. The Tajima’s *D* method was applied to genes with at least five SNPs in the two main populations (Thailand 4,673 (91.0%) and South America 3,549 (70.0%) genes). The majority of Tajima’s *D* values were negative (Thailand 90.2%; South America 64.4%), reinforcing the presence of an excess of low frequency and singleton polymorphisms, potentially due to population expansion in the recent past or purifying selection. For Thailand, we identified 398 (8.5%) genes with positive Tajima’s *D* values, of which 14 were in excess of 2.5 and potentially under balancing selection (**Table 3**). Similarly, for South America, of the 1,260 (35.5%) values that were positive, 12 were in excess of 2.5 (**Table 3**). The loci under potential balancing selection in both populations encode proteins with predominantly roles surface proteins (e.g. MSPs) and antigens. The majority of the 26 genes identified in this study have had positive indices of balancing selection in previous studies **[37, 38]**, or have orthologues in *P*. *falciparum* **[18].**

**Table 3:** Genetic regions under potential balancing selection pressure in South America (SA) and Thailand (T)*.

Chr.	Gene Start	Gene End	Tajima's *D*	Gene**	Annotation	Population
1	521978	527387	3.265	*PVX_088235*	ferlin, putative	SA
3	19187	30715	14.166, 7.134	*PVX_001080*	hypothetical protein, conserved	SA, T
4	265018	267216	2.928	*PVX_002785*	ATP-dependent acyl-CoA synthetase	T
4	562755	566374	4.871, 6.085	*PVX_003840*	serine-repeat antigen 3 (SERA)	SA, T
4	567313	571093	4.944	*PVX_003835*	serine-repeat antigen 1 (SERA)	T
4	572172	575852	3.36	*PVX_003830*	serine-repeat antigen 5 (SERA)	T
4	596283	600192	4.224	*PVX_003805*	serine-repeat antigen (SERA), putative	SA
5	1297808	1301010	3.279	*PVX_090285*	Pvstp1, putative	T
5	1345372	1354047	15.907	*PVX_090325*	reticulocyte binding protein 2c (RBP2c)	T
5	1358748	1360820	5.762	*PVX_090330*	reticulocyte binding protein 2 (PvRBP-2)	T
7	1157742	1162997	5.593, 4.124	*PVX_099980*	merozoite surface protein 1 (MSP1)	SA, T
9	6424	7811	4.195	*PVX_090835*	hypothetical protein	T
10	22046	23460	2.925	*PVX_079700*	hypothetical protein, conserved	T
10	65101	69250	2.793	*PVX_079750*	hypothetical protein, conserved	T
10	1187639	1188909	5.206	*PVX_097760*	60S ribosomal protein L31, RPL31	SA
10	1218512	1221845	2.939,5.585	*PVX_097720*	merozoite surface protein 3 (MSP3.10)	SA, T
10	1272354	1274193	3.869	*PVX_097660*	4-diphosphocytidyl-2-C-methyl- kinase, IspE	SA
10	1306384	1308153	5.991	*PVX_097600*	hypothetical protein, conserved	SA
12	751041	752204	5.578	*PVX_082710*	hypothetical protein	SA
13	37121	59181	3.876	*PVX_084160*	dynein heavy chain, putative	SA
13	128618	131751	4.099	*PVX_084260*	hypothetical protein, conserved	SA
14	3044644	3046339	2.773	*PVX_101575*	hypothetical protein, conserved	T

* Tajima’s *D* > 2.5

** at least 5 SNPs per gene.

### Population structure and evidence of differing directional selection in populations

**Fig 2:**
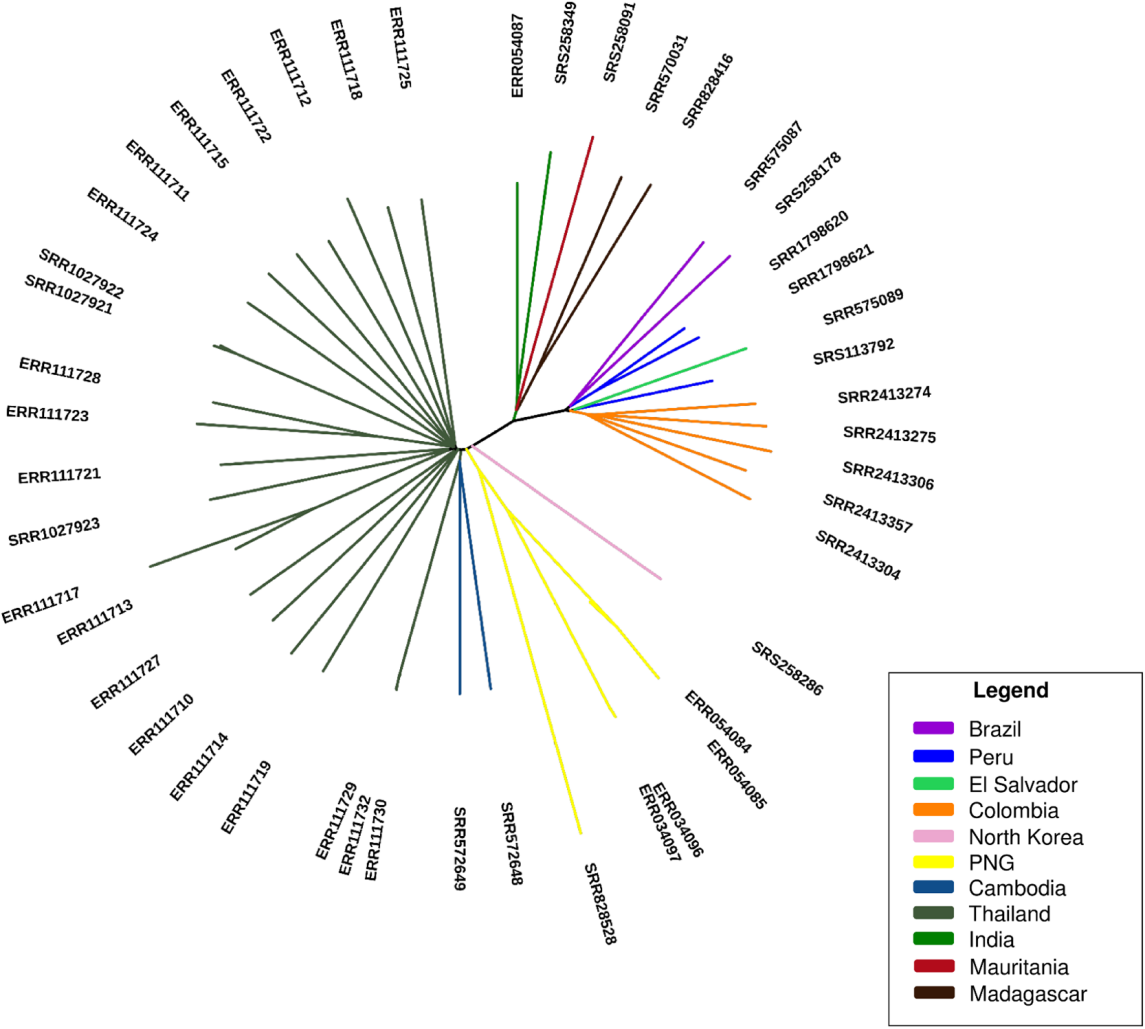
Population structure analysis based on 219,288 SNPs shows clustering by continent.

Both a principal component and a neighbourhood joining tree analysis (**Fig 2, S4 Fig**) revealed clustering by continent, in keeping with similar *P*. *falciparum* analyses **[18, 19].** The across population long-range haplotype method (*Rsb* implementation) was applied to compare Thailand to the South American population, to identify regions potentially under recent directional selection (**Table 4**). We detected several loci including at multidrug resistance-associated protein *MRP1 (PVX_097025)*, and the CCR4-associated factor 1 (*CAF1*, *PVX_123230*) located within 20kb of *DHPS* (associated with resistance to sulfadoxine **[14]**). Five non-synonymous mutations were identified in the *DHPS* gene (M616T, P553A, P383A, P382R, P382A), with evidence that the P383A has driven toward fixation across all geographical regions. Except for mutation in codon 616, all the others have been previously reported **[39–42].** The *DHFR* gene, associated with resistance to pyrimethamine (part of the SP drug combination), exhibited elevated *Rsb* (>3). Seven non-synonymous mutations were identified, including the previously described S58R and S117N **[42–44]** that were fixed across populations, and F57I/L and T61M **[44–46]** that were absent from South America (**S3 Table**). No evidence was observed of a hard sweep around the *MDR1* copy number gene. However, nine non-synonymous SNP mutations were identified, five of which have been reported previously. These included the fixed alleles T958M and M908L, F1076L at high frequency across populations, and G698S and S513R absent from South America **[41–44]**. There was no evidence of a sweep around the *P*. *vivax* orthologues of the *falciparum* chloroquine related *CRT* (*pvcrt-o*, *PVX_087980*) or GTP cyclohydrolase I folate pathway (*GTPCH*, *PVX_123830*) genes. No common non-synonymous mutations were identified within the *CRT* gene, while 7 low frequency non-synonymous SNPs where identified in the *GTPCH* locus.

**Table 4:** Regions under directional selective pressure between Thailand and South America *.

Chr	Position/Range	*Rsb*	Gene	Annotation
2	145708–151606	11.80	.	**Promoter region MRP1**
2	154067–158122	5.230	***PVX_097025***	**multidrug resistance-associated protein 1, MRP1**
2	175191–176803	3.717	*PVX_081215*	hypothetical protein, conserved
4	91457	5.879	*PVX_002550*	hypothetical protein, conserved
4	607568–607837	4.457	*PVX_003795*	serine-repeat antigen (SERA)
4	629831–630120	5.205	*PVX_003770*	merozoite surface protein 5
5	1132736	10.20	*PVX_090105*	holo-[acyl-carrier-protein] synthase, putative (ACPS)
5	964771	3.624	***PVX_089950***	**bifunctional dihydrofolate reductase-thymidylate synthase, DHFR-TS**
6	199049–199165	4.703	PVX_001850	hypothetical protein
6	605656–608119	3.788	PVX_111260	hypothetical protein, conserved
6	635433–635539	4.406	*PVX_111220*	RNA helicase, putative
6	661816	6.048	***PVX_111180***	**28 kDa ookinete surface protein, (P28)**
7	1396929–1396961	4.708	.	Promoter region PVX_086903
7	1397181	4.700	*PVX_086903*	Plasmodium exported protein, unknown function
8	219359–220251	5.257	*PVX_094405*	hypothetical protein, conserved
8	1417014–1417038	4.406	PVX_119515	hypothetical protein, conserved
8	1533222	3.214	*PVX_119360*	hypothetical protein
9	419318–419619	4.971	.	Promoter region PVX_091307
9	920056–920166	4.676	*PVX_091880*	hypothetical protein, conserved
9	1048990	3.304	*PVX_092040*	geranylgeranyl pyrophosphate synthase (GGPPS)
9	1229833	3.296	*PVX_092275*	apical membrane antigen 1 (AMA1)
10	1251585–1257251	7.094	.	Promoter region MSP3.2
10	1257754–1257815	6.617	*PVX_097675*	merozoite surface protein 3 (MSP3.2)
11	1517269	3.234	*PVX_113775*	6-cysteine protein (P12)
11	1223546–1223790	3.816	.	Promoter region PVX_114125
11	1383108–1383155	6.010	*PVX_113925*	hypothetical protein, conserved
12	286960	3.227	***PVX_083240***	**6-cysteine protein (P47)**
13	141889–142286	5.680	*PVX_084280*	hypothetical protein, conserved
13	620154–620261	5.922	.	Promoter region PVX_084770
13	731328–792522	5.375	*PVX_084860*	hypothetical protein, conserved
13	1034635–1034718	4.101	PVX_085235	hypothetical protein
13	1042774	5.406	*PVX_085245*	hypothetical protein, conserved
13	1553113	4.126	*PVX_085835*	hypothetical protein, conserved
14	1231525–1231528	6.056	***PVX_123205***	**CCR4-associated factor 1, (CAF1)**
14	1429874	3.903	*PVX_123415*	adrenodoxin-type ferredoxin, putative

* *Rsb* > 3; genes in bold refer to loci related with mosquito life stages of the parasite or drug-resistance.

The *Rsb* analysis also revealed loci associated with the diversity of vectors, including the *P28* (*PVX_111180*) gene expressed in the surface of the ookinete stage during the mosquito part of the life cycle, *pv47* (*PVX_083240*) and *pv48/45* (*PVX_083235*) involved in the transmission of the parasite. There are continental-specific *pv47* and *pv48/45* SNPs (and haplotypes) as previously found **[47, 48]**, consistent with the presence of different species of mosquito in each the regions **[49],** resembling a similar pattern found in *P*. *falciparum* **[50].**

### Towards molecular barcoding of *P*. *vivax*

The development of molecular barcode for *P*. *vivax* could ultimately assist with surveillance and disease control. Previous work **[51]** has described a 42 SNP barcode to classify geographically *P*. *vivax* across 7 countries. Across the 46 isolates analysed here, we found 3 SNPs in the barcode to be either non-segregating or not passing quality control filtering. Use of the remaining 39 SNPs led to imperfect clustering by continent (**S4 Table, S5 Fig**). Application of the *F*_*ST*_ population differentiation metric identified SNPs driving the observed differences between Thailand, South America and other populations (**S5 Table**). These SNPs occurred in drug resistance loci, including *MRP1 (PVX_097025*), *DHPS* (*PVX_123230*) and *UBP1* (*PVX_081540*) (all *F*_*ST*_ > 0.72), and in close proximity (e.g. *PVX_089960* within 8kb of *DHFR)*. Population differentiation due to genetic diversity in drug resistant loci is also observed in *P*. *falciparum* **[18,19]**.

Previous work has proposed the mitochondria and apicoplast organellar genomes as candidate regions for a barcode **[29].** Genotyping of organellar markers would benefit from greater copy number and coverage as well as highly conserved sequences **[29]**. Eight markers across five apicoplast genes could differentiate Thai and Southeast Asian samples from the other isolates, and two non-genic markers were found to be exclusive to South America (all *F*_*ST*_>0.7, **S6 Table**). No informative mitochondrial markers were identified (all *F*_*ST*_<0.7). Further, as the organelle genomes are known to be highly conserved between *Plasmodia* species, when comparing a set of *P*. *falciparum* geographical markers **[26]** to *P*. *vivax* sequences, we found evidence of positions close in the sequence. Two of the samples (ERR020124 and SRR828528) had a high density of mixed calls in the organellar genomes, in this case, a signature of *P*. *falciparum* overlaying onto *P*. *vivax* (**S6 Fig**). In general, this density signature is indicative of a co-infection of *P*. *vivax* with another *Plasmodium spp*. By comparing the sequencing reads to the *Plasmodium knowlesi* reference genome **[52],** there was no evidence of any *vivax* and *knowlesi* co-infections. However, the presence of a unique triallelic SNP reinforces the potential for an organellar inter-plasmodia species barcode (**S6 Fig**).

## Discussion

Several studies have previously described the genomic diversity of *P*. *vivax* populations using whole genome data, but with low sample sizes. Recently, two papers using a combined collection of over 400 isolates from 17 countries described major genomic diversity in *Plasmodium vivax* **[6, 7]**. Here we analysed a complementary collection of 46 high quality isolates spanning 10 countries across 4 continents in order to position them within the context of this new work. As expected we confirmed that *P*. *vivax* genomic diversity is greater compared to *P*. *falciparum*, and even at a relatively low sample size, the samples clustered geographically. We reveal a wider genomic distance between South American and Southeast Asian continents than observed between *P*. *falciparum* African and Southeast Asian populations **[6, 18, 19]**, highlighted by the greater and more uniform distribution of SNPs with a high *F*_*ST*_ across the genome. Hotspots of selection pressure were identified, including the previously reported *MRP1*, *DHPS* **[14]** and other putative drug resistance genes, as well as several loci related with the mosquito stage of the parasite life cycle. The latter observation is consistent with recent work **[6,7]** and the presence of different *Anopheles* species across continents. We identified structural variants, including extra copies and deletions in the promoter region of the *MDR1* gene, a locus associated with multiple antimalarial drugs **[14].** We also confirmed the duplication in the Duffy binding protein gene (*PvDBP*) in a Madagascan sample, and detected it in Thai isolates. This duplication has been found in parasites from several regions in Africa, South America and Asia **[6,37].** Many of these locations are areas where Duffy-negative individuals make up >45% of the population. However other regions like Cambodia do not present Duffy-negative individuals **[53]**. It has been theorized that the duplication allows the parasite to infect Duffy negative individuals **[53]**, however more research is needed in this area.

Microsatellite genotyping has been used previously to cluster geographically *P*. *vivax* isolates, and together with antigen genotyping identify mixed infections and extent of transmission, used as the basis of genetic epidemiology. In comparison, whole genome sequencing provides a higher specificity in the application of geographical clustering **[51].** While other studies have focused on creating a barcode using the nuclear genome **[51]**, we also considered organelle genomes (mitochondrion and apicoplast), which are more stable over time, do not undergo recombination and are co-inherited **[29]**. The analysis revealed organellar markers that are potentially Southeast Asian and South American specific, and others that highlighted the presence of multi-species mixed infections. The sequencing of large numbers of isolates, beyond currently published samples sizes, will be required to establish robust intra- and inter-species organellar-based barcode. Such large-scale datasets across multiple regions will also serve to identify the high genomic diversity that lies within and between *P*. *vivax* populations, which could be exploited for biological insights, including elucidating drug resistance and invasion mechanisms, and ultimately measures of disease control.

## Conclusion

This study has shown that genomic diversity that lies within and between *P*. *vivax* populations can be used to elucidate potential drug resistance and invasion mechanisms, as well as facilitate the molecular barcoding of the parasite for surveillance applications.

## Supporting information

S1 Fig **Structural variants located around the *MDR1* gene (chromosome 10) in the Thailand population; (i)** a sample without a copy number variant or deletion (even coverage), **(ii)** a major deletion in the promoter region of the gene (n = 7); **(iii)** duplication of ~35kb (position 351kbp to 389kbp, n = 1); and (iv) a combination of both structural variants **(ii)** and **(iii)**, including two copies, one with the deletion in the promoter and another copy with a complete promoter (n = 4, Thailand). The horizontal dashed line is average chromosomal coverage and the red outline encloses the promoter region of the *MDR1* gene.
(TIFF)

S2 Fig Intra-population evidence of directional selective pressure (iHS*) a) Thailand **b)** South America. * *iHS* integrated haplotype score; see **Table 1**and **Table 2**for a summary of the hits.
(TIFF)

S3 Fig Principal component analysis based on 225k SNPs reveals strong clustering of isolates by continent. (PNG)

S4 Fig Identifying regions under directional selective pressure between Thailand and South America. Blue line: *|Rsb|* > 3 (P<0.003); Red line represents a human GWAS cut-off; see **Table 4**for a summary of the hits.
(PNG)

S5 Fig Principal component analysis based on the previously characterised 42 barcoding SNPs* does not reveal strong population clustering. * SNPs and genotypes are shown in **S4 Table**
(PNG)

S6 Fig Signatures of a mixed species infection based on heterozygous calls in mitochondrial markers (positions: 3,736–3,935bp). (PNG)

S1 Table The 46 study isolates. (DOCX)

S2 Table The SNPs. (DOCX)

S3 Table Non-synonymous mutations in candidate genes. (DOCX)

S4 Table Previously characterised 42 barcoding SNPs* in the 46 study isolates. (DOCX)

S5 Table Sites of population differentiation between Thailand and South America. (DOCX)

S6 Table Population informative apicoplast variants. (DOCX)
