# Supplementary figures and images for: Genomic variation in Plasmodium vivax malaria reveals regions under selective pressure
Source: PLoS One. 2017 May 11;12(5):e0177134. doi: 10.1371/journal.pone.0177134 (PMC5426636; doi:10.1371/journal.pone.0177134)

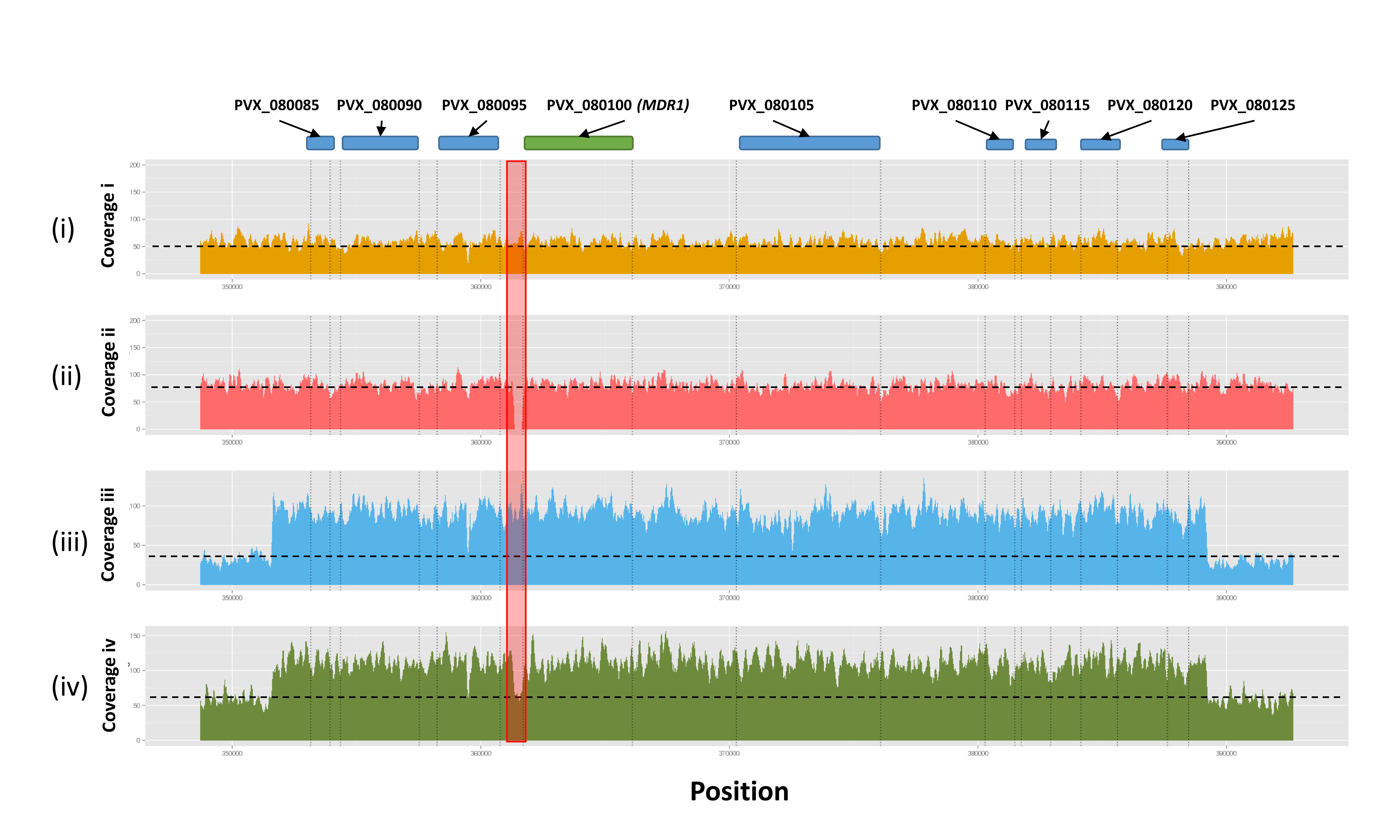

Supplement: S1 Fig — Structural variants located around the MDR1 gene (chromosome 10) in the Thailand population; (i) a sample without a copy number variant or deletion (even coverage), (ii) a major deletion in the promoter region of the gene (n = 7); (iii) duplication of ~35kb (position 351kbp to 389kbp, n = 1); and (iv) a combination of both structural variants (ii) and (iii), including two copies, one with the deletion in the promoter and another copy with a complete promoter (n = 4, Thailand). The horizontal dashed line is average chromosomal coverage and the red outline encloses the promoter region of the MDR1 gene. (TIFF) [file pone.0177134.s001.tiff]

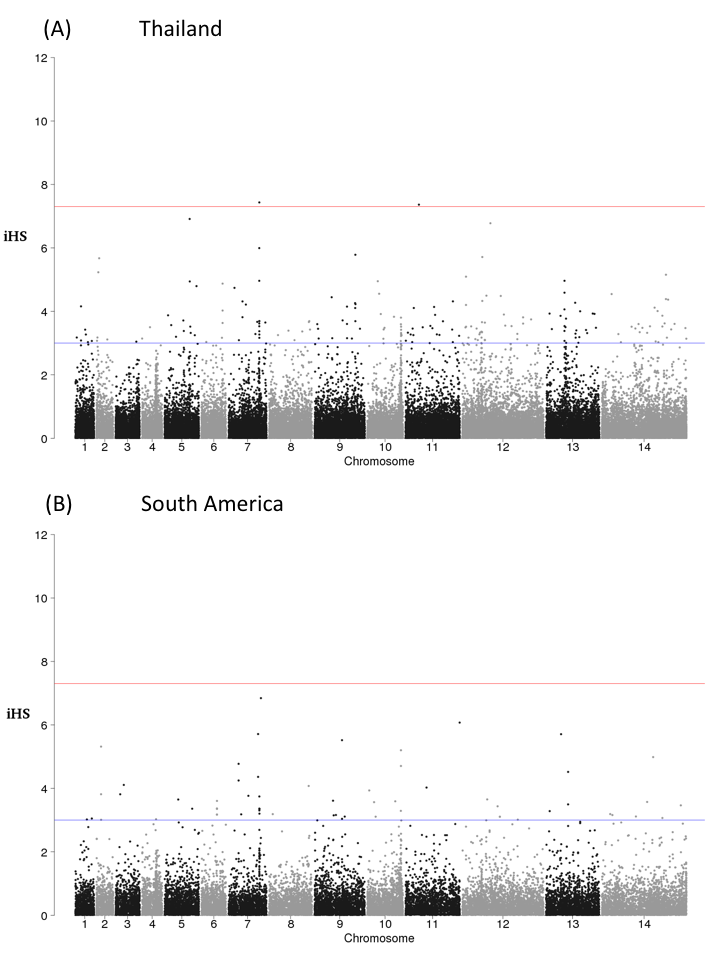

Supplement: S2 Fig — Intra-population evidence of directional selective pressure (iHS*) a) Thailand b) South America. * iHS integrated haplotype score; see Table 1and Table 2for a summary of the hits. (TIFF) [file pone.0177134.s002.tiff]

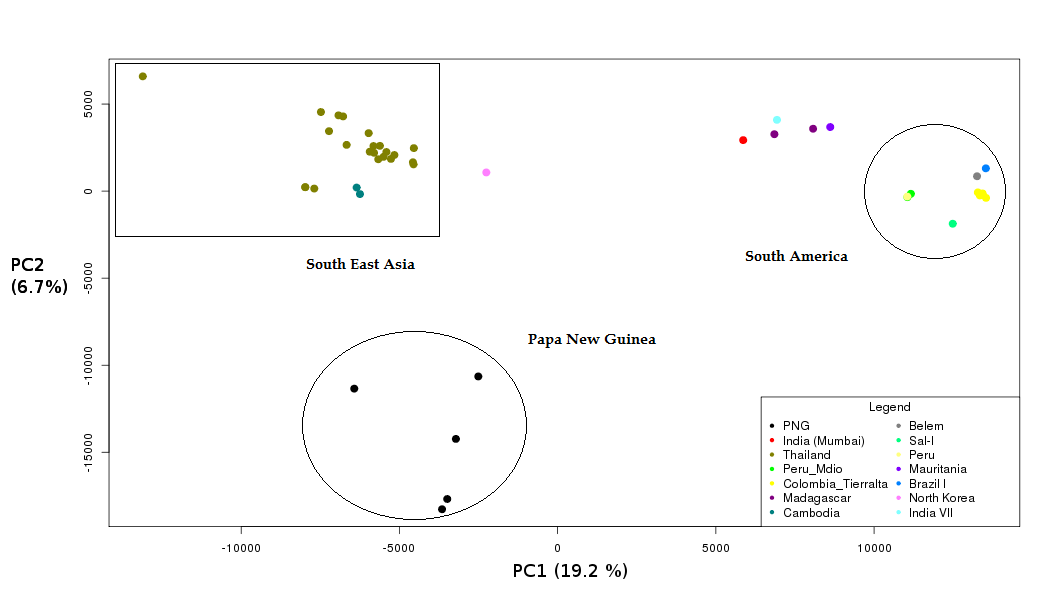

Supplement: S3 Fig — (PNG) [file pone.0177134.s003.png]

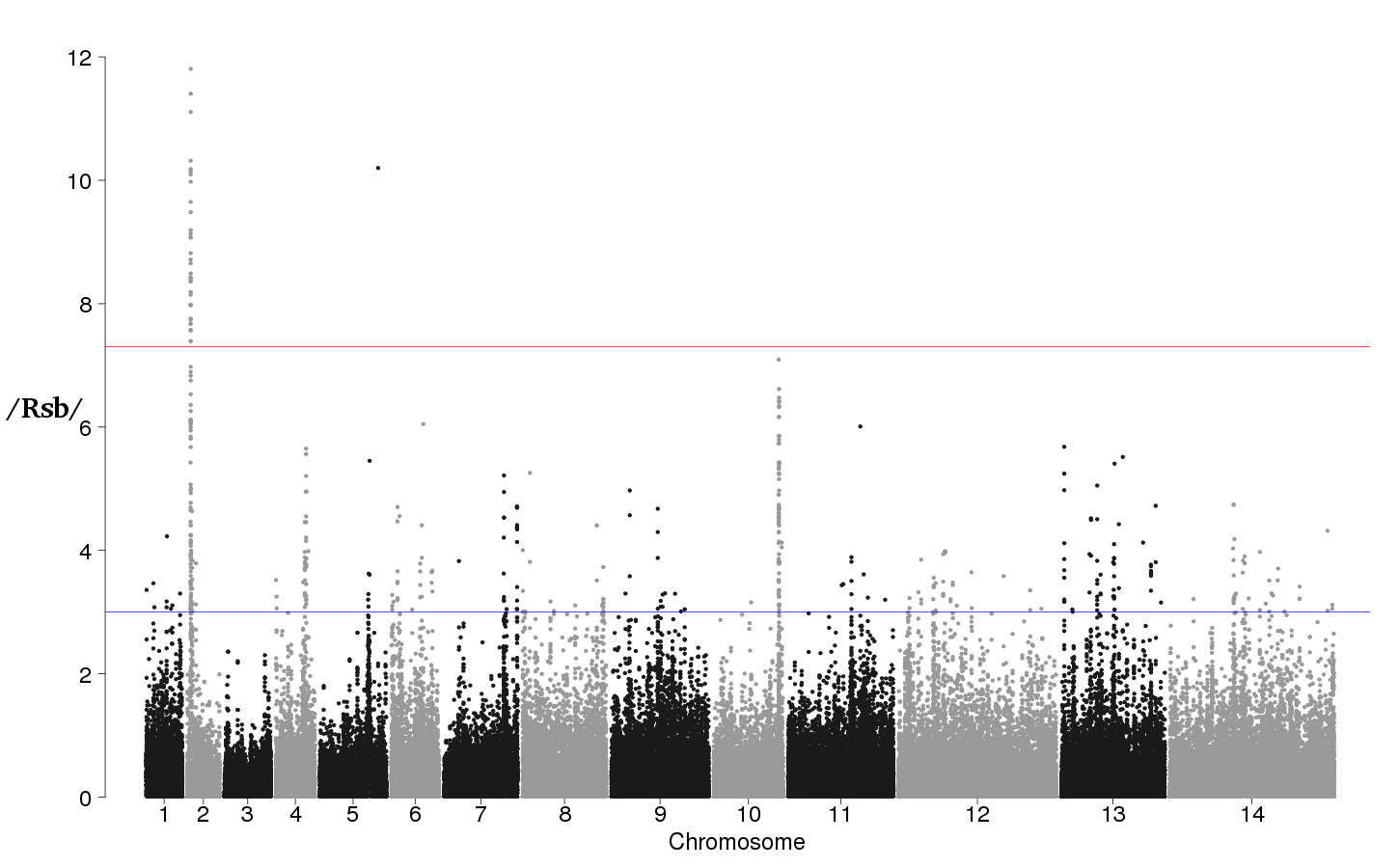

Supplement: S4 Fig — Blue line: |Rsb| > 3 (P<0.003); Red line represents a human GWAS cut-off; see Table 4for a summary of the hits. (PNG) [file pone.0177134.s004.png]

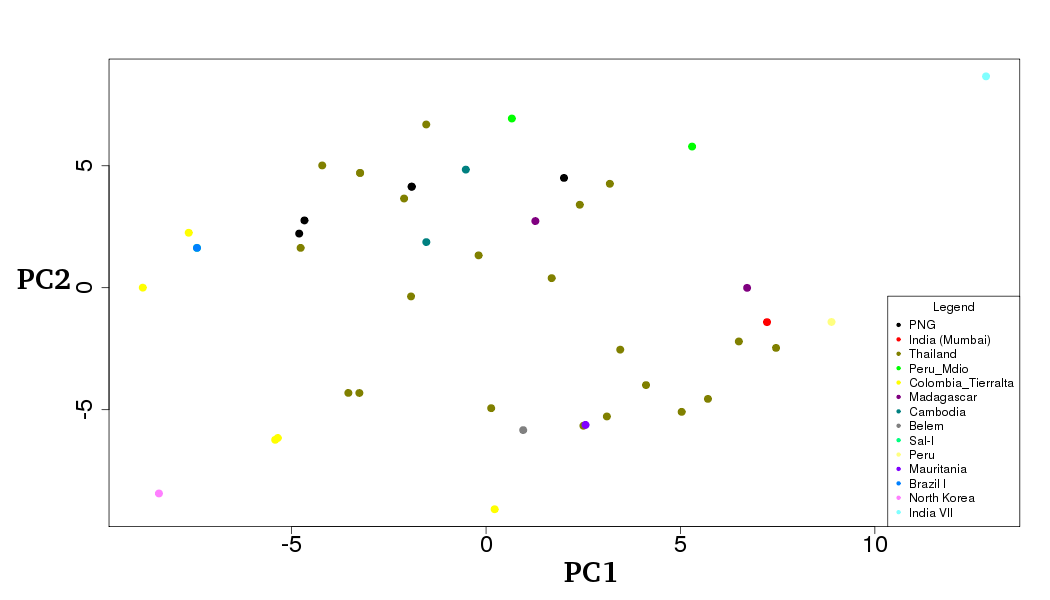

Supplement: S5 Fig — * SNPs and genotypes are shown in S4 Table (PNG) [file pone.0177134.s005.png]

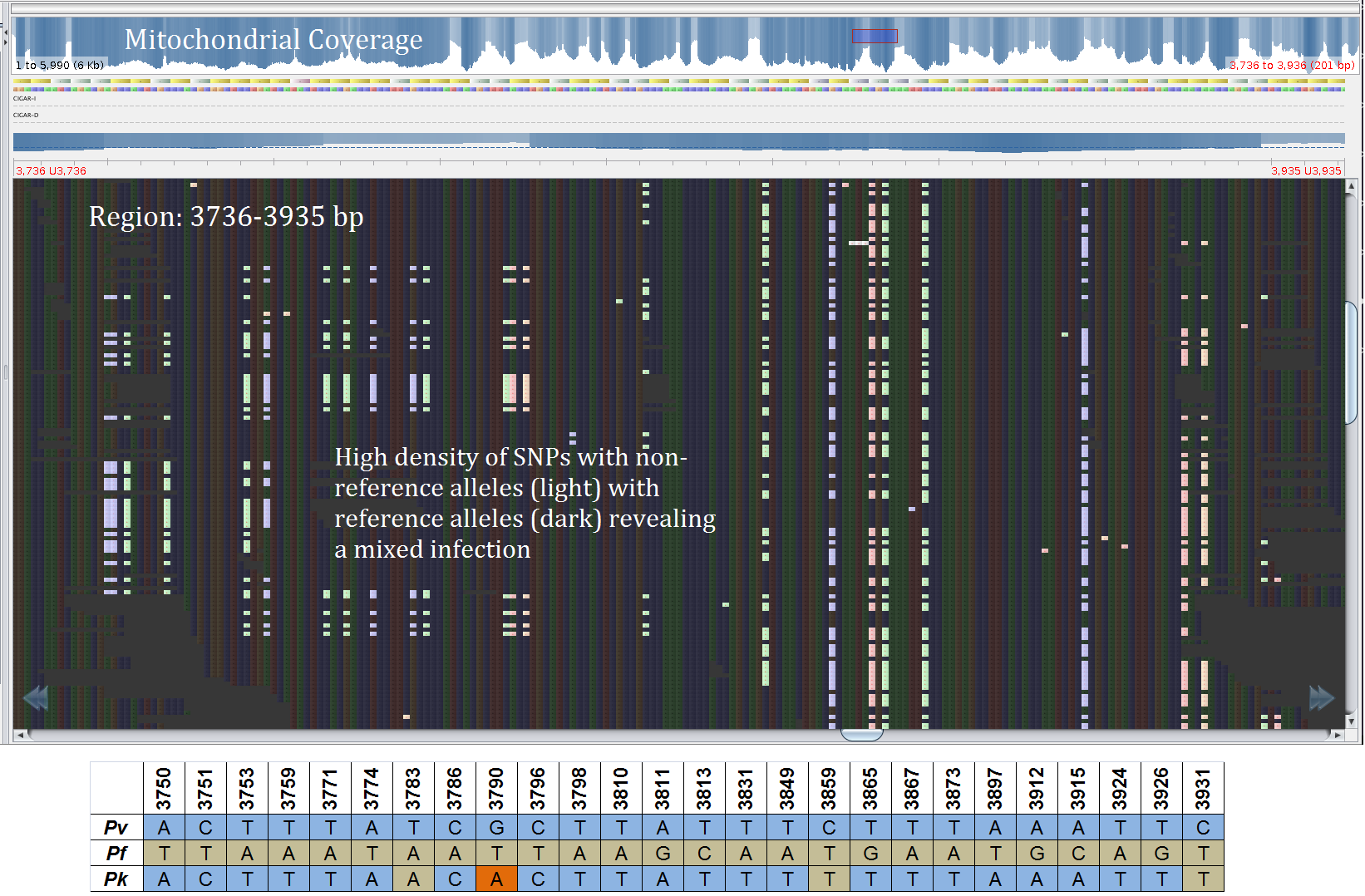

Supplement: S6 Fig — (PNG) [file pone.0177134.s006.png]

**S4 Table**

**Previously characterised 42 barcoding SNPs* in the 46 study isolates**


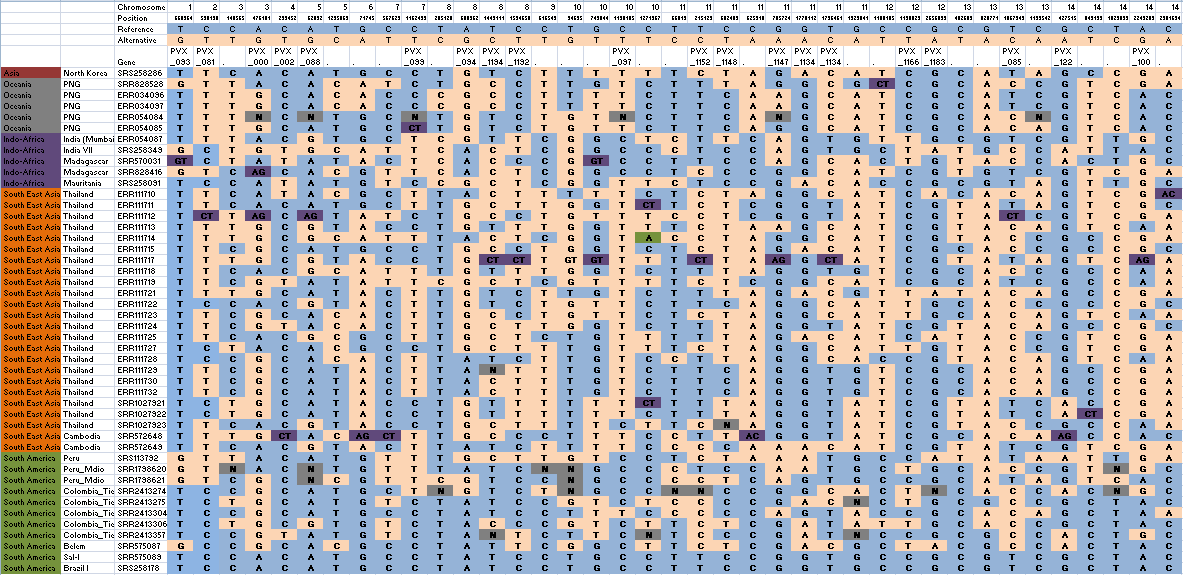


* from reference **[51]**

Supplement: S4 Table — (DOCX) [file pone.0177134.s010.docx]
