# Supplementary material for: Genomic variation in Plasmodium vivax malaria reveals regions under selective pressure
Source: PLoS One. 2017 May 11;12(5):e0177134. doi: 10.1371/journal.pone.0177134 (PMC5426636; doi:10.1371/journal.pone.0177134)
Supplement: S1 Table — (DOCX) [file pone.0177134.s007.docx]

**S1 Table: The 46 study isolates**

| **Country** | **Sample** | **Mean coverage** | **SNPs** |
| --- | --- | --- | --- |
| Cambodia* | SRR572648 | 359.4 | 29,791 |
| Cambodia | SRR572649 | 1082.4 | 29,490 |
| Thailand | ERR111710 | 59.8 | 29,326 |
| Thailand* | ERR111711 | 121.2 | 30,494 |
| Thailand* | ERR111712 | 93.0 | 31,933 |
| Thailand* | ERR111713 | 86.7 | 29,977 |
| Thailand | ERR111714 | 107.5 | 29,945 |
| Thailand* | ERR111715 | 81.0 | 29,632 |
| Thailand* | ERR111717 | 34.5 | 37,674 |
| Thailand | ERR111718 | 94.2 | 30,324 |
| Thailand | ERR111719 | 91.1 | 29,856 |
| Thailand* | ERR111721 | 30.5 | 28,280 |
| Thailand | ERR111722 | 50.2 | 29,370 |
| Thailand* | ERR111723 | 42.1 | 31,034 |
| Thailand | ERR111724 | 59.9 | 30,278 |
| Thailand | ERR111725 | 62.8 | 29,966 |
| Thailand | ERR111727 | 78.1 | 29,672 |
| Thailand* | ERR111728 | 55.7 | 29,773 |
| Thailand* | ERR111729 | 22.7 | 28,504 |
| Thailand | ERR111730 | 86.0 | 30,105 |
| Thailand | ERR111732 | 75.1 | 30,054 |
| Thailand* | SRR1027921 | 63.0 | 30,600 |
| Thailand | SRR1027922 | 104.9 | 30,180 |
| Thailand* | SRR1027923 | 60.7 | 28,949 |
| North Korea | SRS258286 | 2084.2 | 27,722 |
| Brazil I | SRS258178 | 1173.8 | 23,100 |
| Belem | SRR575087 | 629.0 | 22,491 |
| Sal-I | SRR575089 | 31.5 | 108 |
| Peru Mdio | SRR1798620 | 25.7 | 13,151 |
| Peru Mdio | SRR1798621 | 32.8 | 14,580 |
| Peru | SRS113792 | 33.0 | 14,675 |
| Colombia Tierralta* | SRR2413274 | 14.9 | 13,367 |
| Colombia Tierralta* | SRR2413275 | 24.2 | 19,812 |
| Colombia Tierralta* | SRR2413304 | 37.0 | 21,339 |
| Colombia Tierralta* | SRR2413306 | 48.6 | 20,973 |
| Colombia Tierralta* | SRR2413357 | 15.2 | 15,559 |
| Mauritania | SRS258091 | 1703.9 | 28,922 |
| Madagascar* | SRR570031 | 456.6 | 28,077 |
| Madagascar* | SRR828416 | 330.7 | 28,597 |
| Papua New Guinea* | SRR828528 | 54.2 | 36,630 |
| Papua New Guinea | ERR034096 | 136.8 | 30,097 |
| Papua New Guinea | ERR034097 | 62.1 | 29,339 |
| Papua New Guinea | ERR054084 | 14.6 | 18,760 |
| Papua New Guinea* | ERR054085 | 54.9 | 30,004 |
| India (Mumbai) | ERR054087 | 21.4 | 25,098 |
| India VII | SRS258349 | 501.0 | 28,035 |

* evidence of polyclonality using the *estMOI* method [24]
