# Supplementary material for: Genomic variation in Plasmodium vivax malaria reveals regions under selective pressure
Source: PLoS One. 2017 May 11;12(5):e0177134. doi: 10.1371/journal.pone.0177134 (PMC5426636; doi:10.1371/journal.pone.0177134)
Supplement: S2 Table — (DOCX) [file pone.0177134.s008.docx]

**S2 Table**

**The SNPs**

| **Genome** | **Size (bp)** | **Total SNPs** | **Non-Coding** | **Coding** | **Non-Syn.** | **Syn.** | **Intronic** |
| --- | --- | --- | --- | --- | --- | --- | --- |
| Nuclear | 22,621,100 | 219,288 | 107,687 (49%) | 111,601 (51%) | 56,916 (51%) | 35,812  (32%) | 18,873 (17%) |
| Apicoplast | 29,093 | 176 | 53 (30%) | 123 (70%) | 53 (43%) | 70 (57%) | 0  (0%) |
| Mito. | 5,990 | 23 | 16 (70%) | 7  (30%) | 6 (86%) | 1 (14%) | 0  (0%) |
| Total | 22,656,183 | 219,487 | 107,756 (49%) | 111,731 (51%) | 56,975 (51%) | 35,883 (32%) | 18,873 (17%) |

Syn. synonymous
