# Supplementary material for: Genomic variation in Plasmodium vivax malaria reveals regions under selective pressure
Source: PLoS One. 2017 May 11;12(5):e0177134. doi: 10.1371/journal.pone.0177134 (PMC5426636; doi:10.1371/journal.pone.0177134)
Supplement: S3 Table — (DOCX) [file pone.0177134.s009.docx]

**S3 Table**

**Non-synonymous mutations in candidate genes**

| Gene | Chr. | Position | Ref | Alt | Coding Change | Thailand (n=22) | S.America (n=11) | Others (n=13) |
| --- | --- | --- | --- | --- | --- | --- | --- | --- |
| *GTPCH* | 14 | 1825110 | G | C | L38F | 3 | 0 | 0 |
| *GTPCH* | 14 | 1825322 | G | A | G109D | 1 | 1 | 0 |
| *GTPCH* | 14 | 1825574 | G | T | R193I | 2 | 0 | 0 |
| *GTPCH* | 14 | 1825654 | G | A | E220K | 1 | 1 | 0 |
| *GTPCH* | 14 | 1826170 | G | A | G392S | 1 | 1 | 0 |
| *GTPCH* | 14 | 1826180 | C | G | A395G | 2 | 0 | 0 |
| *GTPCH* | 14 | 1826216 | C | T | A407V | 2 | 0 | 0 |
| *DHFR* | 5 | 964758 | T | C,A | F57L,F57I | 18 | 0 | 2 |
| *DHFR* | 5 | 964760 | C | A | F57L | 18 | 0 | 0 |
| *DHFR* | 5 | 964761 | A | C | S58R | 0 | 3 | 0 |
| *DHFR* | 5 | 964763 | C | A,G | S58R,S58R | 22 | 7 | 10 |
| *DHFR* | 5 | 964771 | C | T | T61M | 18 | 0 | 2 |
| *DHFR* | 5 | 964939 | G | C,A | S117T,S117N | 22 | 10 | 9 |
| *DHFR* | 5 | 965106 | A | C | I173L | 0 | 2 | 0 |
| *DHPS* | 14 | 1257156 | A | G | M616T | 0 | 0 | 2 |
| *DHPS* | 14 | 1257346 | G | C | P553A | 18 | 0 | 1 |
| *DHPS* | 14 | 1257856 | G | C | P383A | 22 | 8 | 6 |
| *DHPS* | 14 | 1257858 | G | C | P382R | 7 | 0 | 0 |
| *DHPS* | 14 | 1257859 | G | C | P382A | 0 | 5 | 0 |
| *MDR1* | 10 | 361917 | C | G | K1393N | 4 | 0 | 0 |
| *MDR1* | 10 | 362870 | A | G | F1076L | 14 | 2 | 12 |
| *MDR1* | 10 | 363169 | T | A | Y976F | 5 | 2 | 8 |
| *MDR1* | 10 | 363223 | G | A | T958M | 22 | 10 | 13 |
| *MDR1* | 10 | 363374 | T | G | M908L | 22 | 9 | 13 |
| *MDR1* | 10 | 363514 | G | T | A861E | 3 | 0 | 0 |
| *MDR1* | 10 | 364004 | C | T | G698S | 22 | 0 | 12 |
| *MDR1* | 10 | 364557 | A | T | S513R | 6 | 0 | 3 |
| *MDR1* | 10 | 364598 | C | T | D500N | 0 | 4 | 0 |
| *MDR1* | 10 | 365435 | C | A | V221L | 0 | 3 | 0 |
| *MRP1* | 2 | 154067 | G | A | H1586Y | 2 | 1 | 0 |
| *MRP1* | 2 | 154249 | G | A | T1525I | 0 | 2 | 0 |
| *MRP1* | 2 | 154391 | C | T | V1478I | 22 | 5 | 13 |
| *MRP1* | 2 | 154567 | C | G | G1419A | 1 | 5 | 3 |
| *MRP1* | 2 | 154646 | A | C | Y1393D | 22 | 8 | 13 |
| *MRP1* | 2 | 154979 | A | T | L1282I | 0 | 2 | 0 |
| *MRP1* | 2 | 155204 | G | T | L1207I | 21 | 0 | 1 |
| *MRP1* | 2 | 156107 | G | C | Q906E | 21 | 6 | 11 |
| *MRP1* | 2 | 158047 | G | C | T259R | 22 | 5 | 13 |
| *MRP1* | 2 | 158122 | G | A | T234M | 21 | 0 | 1 |
| *MRP1* | 2 | 158171 | C | A | D218Y | 0 | 0 | 2 |
| *MRP1* | 2 | 158444 | T | C | I127V | 0 | 0 | 2 |
| *MRP1* | 2 | 158717 | T | G | K36Q | 1 | 0 | 1 |
| *MRP1* | 2 | 158795 | G | A | R10C | 0 | 0 | 2 |
| *MRP2* | 14 | 2042200 | G | C | H1960D | 0 | 0 | 2 |
| *MRP2* | 14 | 2043096 | C | G | G1661A | 0 | 0 | 4 |
| *MRP2* | 14 | 2043285 | C | T | S1598N | 0 | 0 | 2 |
| *MRP2* | 14 | 2043838 | G | A | H1414Y | 20 | 0 | 11 |
| *MRP2* | 14 | 2043859 | G | C | Q1407E | 18 | 4 | 13 |
| *MRP2* | 14 | 2044225 | A | G | Y1285H | 2 | 0 | 0 |
| *MRP2* | 14 | 2044528 | G | A | P1184S | 0 | 3 | 0 |
| *MRP2* | 14 | 2044658 | G | T | S1140R | 4 | 0 | 0 |
| *MRP2* | 14 | 2044708 | T | G | T1124P | 1 | 0 | 1 |
| *MRP2* | 14 | 2044749 | A | C | V1110G | 0 | 2 | 4 |
| *MRP2* | 14 | 2044798 | C | A | A1094S | 0 | 5 | 0 |
| *MRP2* | 14 | 2045050 | C | T | V1010M | 22 | 10 | 13 |
| *MRP2* | 14 | 2045101 | C | T | D993N | 2 | 0 | 0 |
| *MRP2* | 14 | 2047069 | A | G | W337R | 4 | 0 | 1 |
| *MRP2* | 14 | 2047224 | A | C | V285G | 2 | 0 | 0 |
| *MRP2* | 14 | 2047233 | C | A | R282M | 22 | 10 | 11 |
| *MRP2* | 14 | 2047269 | G | C | P270R | 3 | 0 | 0 |
| *MRP2* | 14 | 2047816 | C | G | E88Q | 4 | 4 | 4 |
| *MRP2* | 14 | 2047893 | C | T,A | C62Y,C62F | 22 | 0 | 13 |
| *MRP2* | 14 | 2047961 | C | T,G | K39N,Syn | 6 | 0 | 7 |
| *P47* | 12 | 286327 | C | A | F22L | 22 | 1 | 12 |
| *P47* | 12 | 286331 | T | C | F24L | 22 | 0 | 12 |
| *P47* | 12 | 286340 | A | G | K27E | 22 | 1 | 12 |
| *P47* | 12 | 286431 | G | C | S57T | 3 | 0 | 4 |
| *P47* | 12 | 286446 | G | A | S62N | 7 | 0 | 1 |
| *P47* | 12 | 286949 | G | A | V230I | 15 | 0 | 4 |
| *P47* | 12 | 286960 | G | T | M233I | 21 | 0 | 10 |
| *P47* | 12 | 286970 | T | A | F237I | 2 | 0 | 1 |
| *P47* | 12 | 287046 | T | C,A | I262T,I262K | 13 | 1 | 11 |
| *P47* | 12 | 287078 | A | G | I273V | 8 | 0 | 2 |
| *P47* | 12 | 287080 | A | G | I273M | 0 | 0 | 2 |
| *P47* | 12 | 287379 | C | T | A373V | 16 | 1 | 6 |
| *P48/45* | 12 | 289467 | G | A | E35K | 1 | 0 | 2 |
| *P48/45* | 12 | 289995 | C | A | H211N | 22 | 3 | 13 |
| *P48/45* | 12 | 290114 | A | C | K250N | 22 | 4 | 13 |
| *P48/45* | 12 | 290367 | G | T | D335Y | 15 | 0 | 11 |
| *P48/45* | 12 | 290421 | G | C | E353Q | 0 | 3 | 0 |
| *P48/45* | 12 | 290490 | G | A | A376T | 14 | 0 | 10 |
| *P48/45* | 12 | 290617 | A | G | K418R | 22 | 1 | 12 |
