# Supplementary material for: Genomic variation in Plasmodium vivax malaria reveals regions under selective pressure
Source: PLoS One. 2017 May 11;12(5):e0177134. doi: 10.1371/journal.pone.0177134 (PMC5426636; doi:10.1371/journal.pone.0177134)
Supplement: S5 Table — (DOCX) [file pone.0177134.s011.docx]

**S5 Table**

**Sites of population differentiation between Thailand and South America**

| Chr. | Position | FST* | *Gene* | Product |
| --- | --- | --- | --- | --- |
| 1 | 8396 | 0.758 | *PVX_087670* | hypothetical protein, conserved |
| 1 | 137109 | 0.710 | *PVX_087775* | E3 ubiquitin-protein ligase, putative |
| 1 | 422094 | 0.721 | *PVX_088090* | hypothetical protein, conserved |
| 1 | 449291 | 0.710 | *PVX_088130* | AP-3 complex subunit delta, putative |
| 1 | 638670 | 0.668 | *PVX_093565* | hypothetical protein, conserved |
| 2 | 153642 | 0.853 | ***PVX_097025*** | **multidrug resistance-associated protein 1, *MRP1*** |
| 2 | 174990 | 0.726 | *PVX_081215* | hypothetical protein, conserved |
| 2 | 299845 | 0.924 | *PVX_081395* | serine/threonine protein kinase, putative |
| 2 | 408852 | 0.716 | *PVX_081525* | hypothetical protein, conserved |
| 2 | 416092 | 0.710 | ***PVX_081540*** | **ubiquitin carboxyl-terminal hydrolase 1, *UBP1*** |
| 3 | 64629 | 0.694 | *PVX_001050* | SET domain protein, putative (*SET8*) |
| 3 | 466211 | 0.849 | *PVX_000565* | hypothetical protein, conserved |
| 4 | 295743 | 0.716 | *PVX_002815* | hypothetical protein, conserved |
| 4 | 356929 | 0.924 | *PVX_002905* | hypothetical protein, conserved |
| 4 | 481817 | 0.788 | *PVX_003935* | amine transporter, putative |
| 4 | 618245 | 0.668 | *PVX_003780* | hypothetical protein, conserved |
| 4 | 715122 | 0.716 | *PVX_003635* | hypothetical protein, conserved |
| 4 | 812551 | 0.710 | *PVX_003535* | Phist protein (*Pf-fam-b*) |
| 5 | 388029 | 0.767 | *PVX_089215* | serine/threonine protein kinase VPS15, putative (*VPS15*) |
| 5 | 452949 | 0.664 | *PVX_089310* | hypothetical protein, conserved |
| 5 | 591010 | 0.709 | *PVX_089480* | hypothetical protein, conserved |
| 5 | 621120 | 0.726 | *PVX_089510* | D13 protein, putative |
| 5 | 675424 | 0.668 | *PVX_089580* | chaperone protein ClpB1, putative (*ClpB1*) |
| 5 | 972932 | 0.788 | *PVX_089960* | hypothetical protein, conserved |
| 6 | 65601 | 0.709 | *PVX_001690* | Phist protein (*Pf-fam-b*) |
| 6 | 171699 | 0.710 | *PVX_001830* | hypothetical protein, conserved |
| 6 | 246827 | 0.779 | *PVX_001920* | hypothetical protein, conserved |
| 6 | 268713 | 0.783 | *PVX_001950* | hypothetical protein, conserved |
| 6 | 420885 | 0.664 | *PVX_111495* | hypothetical protein, conserved |
| 6 | 521859 | 0.705 | *PVX_111370* | hypothetical protein |
| 6 | 575464 | 0.709 | *PVX_111292* | conserved Plasmodium protein, unknown function |
| 6 | 625419 | 0.779 | *PVX_111230* | hypothetical protein, conserved |
| 7 | 68940 | 0.713 | *PVX_098625* | hypothetical protein, conserved |
| 7 | 154973 | 0.709 | *PVX_098710* | dynein heavy chain, putative |
| 7 | 174254 | 0.674 | *PVX_098712* | high molecular weight rhoptry protein 3, *RhopH3* |
| 7 | 504372 | 0.788 | *PVX_099100* | hypothetical protein, conserved |
| 7 | 568345 | 0.709 | *PVX_099180* | type II NADH:ubiquinone oxidoreductase, putative |
| 7 | 726188 | 0.709 | *PVX_099375* | hypothetical protein, conserved |
| 7 | 1019659 | 0.726 | *PVX_099750* | hypothetical protein, conserved |
| 7 | 1038999 | 0.726 | *PVX_099780* | replication termination factor, putative |
| 7 | 1064692 | 0.730 | *PVX_099820* | hypothetical protein, conserved |
| 8 | 172625 | 0.924 | *PVX_094350* | hypothetical protein, conserved |
| 8 | 573474 | 0.705 | *PVX_094855* | NLI interacting factor-like phosphatase, putative (*NIF4*) |
| 8 | 730101 | 0.705 | *PVX_095010* | ribonucleotide reductase small subunit, putative |
| 8 | 787639 | 0.716 | *PVX_095095* | hypothetical protein, conserved |
| 8 | 829721 | 0.713 | *PVX_095145* | hypothetical protein, conserved |
| 8 | 1502402 | 0.710 | *PVX_119390* | hypothetical protein, conserved |
| 9 | 20038 | 0.664 | *PVX_090840* | hypothetical protein |
| 9 | 77676 | 0.694 | *PVX_090895* | hypothetical protein, conserved |
| 9 | 102780 | 0.716 | *PVX_090925* | protein kinase domain containing protein |
| 9 | 279221 | 0.713 | *PVX_091136* | hypothetical protein, conserved |
| 9 | 659985 | 0.710 | *PVX_091630* | hypothetical protein, conserved |
| 9 | 729619 | 0.709 | *PVX_091700* | circumsporozoite-related antigen, putative (*EXP1*) |
| 9 | 1154029 | 0.769 | *PVX_092185* | hypothetical protein, conserved |
| 9 | 1316937 | 0.713 | *PVX_092370* | hypothetical protein, conserved |
| 10 | 667466 | 0.664 | *PVX_080425* | transporter, putative |
| 10 | 671226 | 0.924 | *PVX_080430* | phosducin-like protein, putative (*PhLP3*) |
| 10 | 800750 | 0.850 | *PVX_080615* | hypothetical protein, conserved |
| 10 | 829185 | 0.708 | *PVX_080660* | RNA pseudouridylate synthase, putative |
| 10 | 973520 | 0.712 | *PVX_098015* | soluble NSF attachment protein (*SNAP*), putative |
| 10 | 975894 | 0.668 | *PVX_098010* | hypothetical protein |
| 10 | 1003754 | 0.779 | *PVX_097980* | transcription factor IIb, putative |
| 11 | 98032 | 0.924 | *PVX_115400* | DNA repair endonuclease, putative (*ERCC4*) |
| 11 | 565497 | 0.849 | *PVX_114865* | T-complex protein 1 subunit delta, putative (*CCT4*) |
| 11 | 920422 | 0.709 | *PVX_114495* | acetyl-CoA synthetase, putative (*ACS*) |
| 11 | 1505435 | 0.710 | *PVX_113790* | hypothetical protein, conserved |
| 11 | 1530963 | 0.726 | *PVX_113750* | eukaryotic translation initiation factor protein |
| 11 | 1686293 | 0.730 | *PVX_113576* | sorting assembly machinery 50 kDa subunit, *SAM50* |
| 11 | 1700734 | 0.674 | *PVX_113560* | hypothetical protein, conserved |
| 11 | 1912448 | 0.668 | *PVX_113325* | mitochondrial chaperone *BCS1*, putative |
| 11 | 1938368 | 0.775 | *PVX_113285* | elongation factor G, putative |
| 12 | 174109 | 0.668 | *PVX_083360* | tyrosine kinase-like protein, putative (*TKL3*) |
| 12 | 212484 | 0.779 | *PVX_083315* | hypothetical protein, conserved |
| 12 | 225457 | 0.712 | *PVX_083310* | translation elongation factor, putative |
| 12 | 339353 | 0.668 | *PVX_083185* | isocitrate dehydrogenase [*NADP*], mitochondrial, *IDH* |
| 12 | 537177 | 0.726 | *PVX_082980* | GPI mannosyltransferase 3, putative (*GPI10*) |
| 12 | 575061 | 0.705 | *PVX_082937* | hypothetical protein, conserved |
| 12 | 890022 | 0.776 | *PVX_082470* | elongation factor Tu, mitochondrial precursor, putative |
| 12 | 1173110 | 0.730 | *PVX_116655* | hypothetical protein, conserved |
| 12 | 1307254 | 0.788 | *PVX_116805* | hypothetical protein, conserved |
| 12 | 1403249 | 0.668 | *PVX_116930* | conserved Plasmodium protein, unknown function |
| 12 | 1528154 | 0.721 | *PVX_117065* | TLD domain-containing protein |
| 12 | 1588169 | 0.850 | *PVX_117145* | transcription factor with AP2 domain(s), *ApiAP2* |
| 12 | 1623826 | 0.790 | *PVX_117175* | dynein beta chain, putative |
| 12 | 1853264 | 0.664 | *PVX_117405* | hypothetical protein, conserved |
| 12 | 2167573 | 0.790 | *PVX_117815* | hypothetical protein, conserved |
| 12 | 2471252 | 0.701 | *PVX_118175* | hypothetical protein, conserved |
| 13 | 307322 | 0.783 | *PVX_084445* | cysteine repeat modular protein 3, putative (*CRMP3*) |
| 13 | 466345 | 0.674 | *PVX_084600* | DnaJ domain containing protein |
| 13 | 503529 | 0.664 | *PVX_084640* | hypothetical protein |
| 13 | 696401 | 0.775 | *PVX_084840* | hypothetical protein, conserved |
| 13 | 725430 | 0.715 | *PVX_084860* | hypothetical protein, conserved |
| 13 | 773964 | 0.783 | *PVX_084925* | hypothetical protein, conserved |
| 13 | 1012549 | 0.705 | *PVX_085205* | ABC transporter G family member 2, putative (*ABCG2*) |
| 13 | 1146760 | 0.783 | *PVX_085390* | hypothetical protein, conserved |
| 13 | 1209407 | 0.726 | *PVX_085490* | glutathione reductase, putative |
| 13 | 1673325 | 0.702 | *PVX_085955* | cytidine diphosphate-diacylglycerol synthase, putative |
| 13 | 1773064 | 0.709 | *PVX_086035* | transcription factor with AP2 domain(s), *AP2-G2* |
| 14 | 129079 | 0.712 | *PVX_121945* | gametocyte associated protein, putative (*GAP*) |
| 14 | 188124 | 0.668 | *PVX_122015* | sodium/hydrogen exchanger 1, putative |
| 14 | 918073 | 0.767 | *PVX_122845* | hypothetical protein, conserved |
| 14 | 1058168 | 0.924 | *PVX_122995* | transporter, putative |
| 14 | 1254772 | 0.668 | *PVX_123225* | hypothetical protein, conserved |
| 14 | 1256701 | 0.668 | ***PVX_123230*** | **hydroxymethylpterin pyrophosphokinase-dihydropteroate synthetase, putative (DHPS)** |
| 14 | 1263307 | 0.668 | *PVX_123240* | DEAD/DEAH box helicase, putative |
| 14 | 1296964 | 0.668 | PVX_123283 | JmjC domain containing protein (*JmjC1*) |
| 14 | 1314634 | 0.836 | *PVX_123300* | hypothetical protein, conserved |
| 14 | 1416599 | 0.770 | *PVX_123395* | *GPI* ethanolamine phosphate transferase 3, *PIGO* |
| 14 | 1963668 | 0.713 | *PVX_124005* | hypothetical protein, conserved |
| 14 | 2432144 | 0.668 | *PVX_100865* | cell cycle control protein, putative |
| 14 | 2759960 | 0.779 | *PVX_101265* | cyclin g-associated kinase, putative |
| 14 | 2957235 | 0.721 | *PVX_101500* | hypothetical protein |
| API | 22771 | 0.726 | ***PVIV_000008700*** | ***sufB*** |

API apicoplast, all *F_ST_* >0.68; **bolded** – known drug resistance loci
