# Supplementary material for: Genomic variation in Plasmodium vivax malaria reveals regions under selective pressure
Source: PLoS One. 2017 May 11;12(5):e0177134. doi: 10.1371/journal.pone.0177134 (PMC5426636; doi:10.1371/journal.pone.0177134)
Supplement: S6 Table — (DOCX) [file pone.0177134.s012.docx]

**S6 Table**

**Population informative apicoplast variants**

| **Chr.** | **Pos** | **Ref. Allele** | **Alt.**  **Allele** | ***F_ST_***  **Thailand vs. other** | ***F_ST_***  **SEA vs. other** | ***F_ST_***  **South America vs. other** | **Gene** |
| --- | --- | --- | --- | --- | --- | --- | --- |
|  |  |  |  |  |  |  |  |
| API | 1,416 | G | A | 0.265 | 0.238 | **1.000** | *.* |
| API | 2,461 | A | G | 0.289 | 0.258 | **1.000** | *.* |
| API | 5,562 | A | G | **0.729** | **0.620** | 0.207 | *RPS8* |
| API | 8,308 | C | T | **0.720** | **0.625** | 0.186 | *RPS7* |
| API | 16,619 | T | C | **0.729** | **0.628** | 0.193 | *.* |
| API | 18,222 | G | A | **0.729** | **0.634** | 0.193 | *rpoC* |
| API | 20,024 | T | C | **0.729** | **0.634** | 0.193 | *rpoB* |
| API | 22,668 | G | A | 0.289 | 0.258 | **1.000** | *.* |
| API | 23,740 | A | C | **0.729** | **0.634** | 0.193 | *sufB* |
| API | 23,829 | C | T | **0.729** | **0.634** | 0.193 | *sufB* |
| API | 26,441 | C | T | **0.729** | **0.634** | 0.193 | *.* |

SEA Southeast Asia
